# Supporting the patients with advanced cancer and their family caregivers: what are their palliative care needs?

**DOI:** 10.1186/s12885-020-07239-9

**Published:** 2020-08-15

**Authors:** Gek Phin Chua, Grace Su Yin Pang, Alethlea Chung Pheng Yee, Patricia Soek Hui Neo, Siqin Zhou, Cindy Lim, Yin Yee Wong, Debra Limin Qu, Fang Ting Pan, Grace Meijuan Yang

**Affiliations:** 1 CEIS (Research & Data), National Cancer Centre Singapore, 11 Hospital Crescent, Singapore, Singapore, 169610 Singapore; 2 Medical Services, Assisi Hospice, Singapore, Singapore; 3 Division of Supportive and Palliative Care, National Cancer Centre Singapore, Singapore, Singapore; 4 Division of Clinical Trails and Epidemiological Sciences, National Cancer Centre Singapore, Singapore, Singapore; 5 Quality department, Assisi Hospice, Singapore, Singapore; 6 Lien Centre Palliative Care, Singapore, Singapore

**Keywords:** Advanced cancer, Cancer patients, Caregivers, Palliative care, Needs, Oncology

## Abstract

**Background:**

The impact and consequences of cancer on the patients and their family caregivers (FCs) are closely intertwined. Caregivers’ burdens can be increased due to the patients’ unmet needs and unresolved problems. Additionally, the caregivers’ unmet needs may adversely affect their own well-being and the patients’ health outcomes. This study aims to determine the palliative care needs and the factors associated with these needs in patients with advanced solid cancer and their FCs.

**Methods:**

In a cross-sectional survey, 599 patients with advanced solid tumours and 599 FCs were recruited from the largest ambulatory cancer centre and the inpatient ward of the largest hospital in Singapore. Determinants of patients’ and FCs’ needs were assessed by the Comprehensive Needs Assessment Tool (CNAT) and CNAT-C respectively. Clinical characteristics of patients were obtained from medical records.

**Results:**

The FCs (median age 51 years) were younger than the patients (median age 62 years), and were mostly female (62.6%) whereas the gender distribution of patients was quite balanced (49.2% male and 50.8% female). Both patients and FCs had “information” and “practical support” in their top three domains of palliative care needs. The second highest domain of needs was “psychological problems” (16.4 ± 21.5) in patients and “health-care staff” (23.4 ± 26.5) in FCs. The item that had the highest need score in “information” domain for both patients and FCs was “financial support for patients, either from government and/ or private organizations”. Under clinical setting, the inpatients (19.2 ± 16.4) and their FCs (26.0 ± 19.0) tend to have higher needs than the outpatients (10.5 ± 12.1) and their FCs (14.7 ± 14.3). In terms of palliative care, higher total CNAT score was observed in both patients (16.6 ± 12.9 versus 13.3 ± 15.2) and their FCs (25.1 ± 18.6 versus 17.7 ± 16.7) who received palliative care. In terms of patients’ KPS scores, patients with lower KPS scores tend to have higher needs.

**Conclusion:**

Overall, the findings confirm that patients with advanced cancer and their FCs have many palliative care needs irrespective of their clinical settings. Initiatives and interventions for the development of a comprehensive support system for both patients with advanced cancer and their FCs are warranted and can be derived from these findings.

## Background

Medical advances in the treatment of cancer have enabled patients diagnosed with advanced cancer to live for a relatively long period. This change in prognosis may bring considerable needs and problems to both patients and caregivers. Physical dysfunction [1] cognitive dysfunction [2, 3], psychological dysfunction [2] and economic, financial and insurance concerns [2, 4, 5] have been identified as long-term consequences of cancer and its treatment.

Family caregivers (FCs) play an integral role in the care and support of cancer patients. They may assume diverse responsibilities during the patient’s disease trajectory providing physical, emotional, social, spiritual and financial support [6, 7]. The long-term process of providing care is physically and psychologically demanding, especially when caring for patients with advanced cancer. The burden of caregiving may manifest in symptoms of sleep difficulties, depression, anxiety, tension, panic, or behaviours that may jeopardize the FC’s health [5, 6, 8–14]. FCs of patients with terminal cancer can experience an even higher burden as the patient’s condition deteriorates and family financial resources become exhausted and physical energy and emotions are drained [15].

The impact and consequences of cancer on the patients and their FCs are closely intertwined. Evidence in literature indicates that the level of caregiver burden can be increased due to the patient’s unmet needs [16]. In addition, the challenges that caregivers experienced are closely related to the well-being of the patients [14]. Unresolved problems or unmet needs of caregivers will not only adversely affect the caregivers’ own well-being [11] and decrease their quality of life [17, 18] but also the patients’ health outcomes [19]. Conversely, the health status of patients may improve due to expertise, confidence, and ability of the caregivers to provide quality care [7].

Despite being a relatively young nation, Singapore has attained a relatively high standard of health. Life expectancy for males is 81 years and females is 85.4 years [20]. Like many developed countries, cancer has been the leading cause of mortality in Singapore. Unlike many developed nations such as the United Kingdom and Australia, the development of palliative care was slow due to the Asian taboos regarding dialogue about death and dying [21]. Palliative care as defined by World Health Organization is “an approach that improves the quality of life of patients and their families facing the problem associated with life threatening illness, through the prevention of suffering by means of early identification and impeccable assessment of pain and other problems, physical, psychosocial and spiritual” [22]. The aim of palliative care is to improve the quality of life (QOL) for individuals with a life-limiting illness and their families [23].

Efforts have been made to expand and improve palliative care in Singapore such as raising public awareness, redesigning healthcare professionals’ education and improving funding by the state. It was only in 2006 that palliative medicine was recognized as a medical specialty in Singapore [21] and is increasingly becoming a part of mainstream medicine and acknowledged as an essential part of the healthcare system. The first hospice with 16 beds was set up by a group of Catholic nuns at the St. Joseph Home in 1985 [21] for dying patients. Over the years, the scope and range of palliative care services have expanded. Palliative care is now being offered in hospitals, hospices and home and is extended to non-cancer patients. There are also plans to introduce palliative care early in the course of a potentially life-limiting illness [24].

In order to provide high quality patient and family centered care, the care needs of both patients and FCs should be comprehensively assessed. Assessing the needs of both patients and their FCs is critical to guide care planning in supporting them to cope. Relatively, few studies have explored the needs of caregivers of cancer patients [12, 13, 15, 18, 25–28] and even fewer studies have investigated these needs directly from the patients and their FCs [15]. Moreover, the needs of patients and their FCs in Singapore may differ from that in other countries because of differing cultural norms and expectations. Only a few studies have evaluated the experience of Singapore caregivers of cancer patients and they are related to burden of care [29], quality of life [30] and unmet needs [17].

The purpose of this study is to determine the palliative care needs and the factors associated with these needs in patients with advanced solid cancer and their FCs; and identify gaps in palliative care services that need to be addressed. To the best of our knowledge, this is the first assessment and analysis of the palliative care needs of both patients with advanced cancer and their FCs in Singapore. The body of knowledge derived from this study would provide an evidence-based approach in the delivery of patient and family centric palliative care. It would further add to the growing literature on the palliative care needs of patients with advanced cancer and their FCs.

## Methods

### Participants, settings and study procedure

An exploratory cross-sectional survey was conducted at the outpatient clinics of the National Cancer Centre Singapore (NCCS, the largest public ambulatory cancer centre in Singapore), and at the inpatient wards of the Singapore General Hospital (the largest hospital in Singapore and the admitting hospital for patients from the NCCS). Eligible patients were first identified by the primary physician or palliative care team the patient was referred to. The trained research coordinator would help the physicians and team to identify eligible patients by pre-screening through the outpatients or inpatient resource list or the referring team contacts the research coordinator when there is an eligible caregiver. The inclusion criteria for patients were: (1) aged 21 years and above (the age of majority), (2) able to understand English or Mandarin, (3) intact cognition, (4) diagnosed with advanced solid tumour that is not under curative treatment, (5) Karnofsky performance status ≥20 and, (6) agreed to participate in this study. Inclusion criteria for caregivers were: (1) aged 21 years and above, (2) able to understand English or Mandarin, (4) family members of patients with advanced solid tumour that is not under curative treatment and, (5) agreed to participate in the study. The patients were asked to identify their primary FC, defined as a relative who provided them with the most assistance in terms of physical caregiving or decision making.

Eligible patients and FCs were assessed for eligibility to participate. Detailed explanation of the study’s purposes and procedures as well as a copy of the Participant Information Sheet was provided to the patient and/or FC prior to obtaining their consent for participation in the study. Eligible participants were informed about the voluntary nature of the study and they were able to withdraw from the study at any time without compromising on the quality of care that would be rendered. Written informed consents were obtained from patients/ and/or their/ FCs. The questionnaires were administered by trained interviewers to eligible patients/ and/or FCs who are not able to read but able to understand their native language verbally in a quiet room at the outpatient clinics or inpatient wards. Clinical information of patients was collected directly from the medical record. The study was conducted from 1 April 2014 to 1 December 2016.

Ethical approval to conduct this study was obtained from the Centralized Institutional Review Board of the Singapore Health Services.

#### Measures

##### Patients

1. Background information form

The background information obtained from the patients included race, nationality, gender, marital status, education, housing type, employment status, tumour site, number of co-morbidities, clinical setting, and whether patients were receiving palliative care (under the care of palliative care physicians/ organizations). Functional status was measured by Karnofsky Performance Status (KPS) index [31].

2. Comprehensive needs assessment tool (CNAT)

Needs were assessed with the comprehensive needs assessment tool (CNAT) for cancer patients. The 59-item questionnaire measure 7 domains of need: (1) information and education (10 items), (2) psychological problems (10 items), (3) healthcare staff (8 items), (4) physical symptoms (12 items), (5) hospital facilities and services (8 items), (6) social and religious/spiritual support (5 items), and (7) practical support (6 items). Items are scored on a 4-point scale of severity according to the level of need: (1) No need help, (2) A little help needed, (3) Moderate help needed and, (4) A lot of help needed. The CNAT has good validity with a reported coefficient of reliability of Cronbach’s alpha of between 0.80 and 0.937 [32].

##### Caregiver’s questionnaire

1. The background information

The background information obtained from the patients’ FCs included race, nationality, gender, marital status, education, housing type, employment status, relationship to patient, caregiving roles, assistance with caregiving, caregiver’s medical condition, clinical setting, and whether patients were receiving palliative care. Functional status of the patients cared for was measured by Karnofsky Performance Status (KPS) index [31].

2. Caregiver needs assessment

We used the 41-item Comprehensive Needs Assessment Tool (CNAT-C) to assess the needs of FCs of cancer patients in 7 domains: (1) health and psychological problems (6 items), (2) family/ social support (5 items), (3) Health-care staff (8 items), (4) information (8 items), (5) religious/spiritual support (2 items), (6) hospital facilities and services (6 items), and (7) practical support (6 items). Each domain contains 2 to 8 items. Items are scored on a 4-point scale of severity according to the level of need: (1) No need help, (2) A little help needed, (3) Moderate help needed and, (4) A lot of help needed. The CNAT-C has good validity with a reported coefficient of reliability of Cronbach’s alpha of between 0.75 and 0.95 [33].

#### Statistical methods

The sample size is estimated to be 600 patients and 600 FCs. In order to compare the mean scores between 2 independent groups (inpatient versus SOC setting; with PC versus without PC) using two-tailed t-test, a total sample size of 574 gives 90% power at 5% significance level, to detect an effect size as small as 0.3 for group size that may vary up to a 2:5 ratio. The estimated sample size is 600 considering there might be a small proportion (< 5%) of participants with incomplete filling of questionnaire.

Data were analysed for the entire cohort of respondents, and by difference in needs between cancer patients and FCs. The differences in needs were further analysed based on clinical significance and across settings (inpatients vs outpatients), care received (with or without PC), and KPS scores. As there is a paucity of local data on palliative care needs in patients with advanced cancer and FCs in Singapore, hence an exploratory comprehensive assessment of needs would add to the body of knowledge that is currently lacking.

Scoring of patients and caregivers needs were performed according to the guidelines set by each questionnaire. For both patients and caregivers, each item in the respective surveys reflects the specific level of needs of patients/caregivers in the previous month by “0 (No need help)”, “1 (A little)”, “2 (Moderately)”, and “3 (A lot)”. A higher score would then indicate higher needs. The subdomain scores are derived by computing the mean score of the questions in the subdomain followed by rescaling the scores to a 0–100 scale. The CNAT total score is derived by computing the mean score of all the CNAT questions followed by rescaling the score to a 0–100 scale.

Demographics and clinical characteristics were summarized using frequency and percentage for categorical variables, and median and range for continuous variables. ANOVA tests were carried out on the subdomain scores to compare the prevalence of needs between patients who received and didn’t receive palliative care (under or not under the care of palliative team/ organisations), among patients with KPS score in the range of 0–40, 50–70 and 80–100, and between patients with outpatient and inpatient clinical setting. Univariable and multivariable regression analysis were performed to assess the association between characteristics and total CNAT scores. Variable selection for multivariable analysis was performed using the backward elimination method, by optimizing the Akaike Information Criterion (AIC).

Statistical significance was assessed by two-sided *p*-value less than 0.05. All statistical analyses were performed using R software (version 3.6.0).

## Results

### Participants characteristics

The data consisted of 599 patients with advanced cancer and 599 FCs recruited. Comparing advanced cancer patients with FCs, the demographics of patients appeared to be different from that of the FCs (Table 1). The FCs (median age 51 years) were younger than the patients (median age 62 years), and were mostly female (62.6%) where the gender distribution of patients was quite balanced (49.2% male and 50.8% female). Only 29.4% of patients were employed while 59.8% FCs were working and caregiving for the patients concurrently. The caregiving role was mainly undertaken by children or spouse (43.7 and 43.1% respectively). Most of the patients (97.2%) and FCs (98.7%) were aware of the cancer diagnosis.

**Table 1:** Sample characteristics

		Patients (***n*** = 599)	Caregivers (***n*** = 599)
Age	Median (Q1,Q3)	62.0 (55.0, 68.0)	51.0 (40.0, 59.0)
	Range	24.0–91.0	21.0–81.0
Clinical Setting	SOC	350 (58.4%)	294 (49.1%)
	Inpatient	249 (41.6%)	305 (50.9%)
Race	Chinese	495 (82.6%)	467 (78.0%)
	Malay	67 (11.2%)	84 (14.0%)
	Indian	25 (4.2%)	37 (6.2%)
	Others	12 (2.0%)	11 (1.8%)
Gender	Male	295 (49.2%)	224 (37.4%)
	Female	304 (50.8%)	375 (62.6%)
Marital Status	Single	78 (13.0%)	122 (20.4%)
	Married	453 (75.6%)	467 (78.0%)
	Divorced	20 (3.3%)	5 (0.8%)
	Widowed	48 (8.0%)	5 (0.8%)
Housing Type	Don’t wish to say	4 (0.7%)	2 (0.3%)
	HDB 1 to 2 rooms	42 (7.0%)	25 (4.2%)
	HDB 3 to 4 rooms	293 (48.9%)	282 (47.1%)
	HDB 5 rooms and above	171 (28.5%)	185 (30.9%)
	Private residential property	89 (14.9%)	105 (17.5%)
Employment Status	Unemployed	423 (70.6%)	241 (40.2%)
	Employed	176 (29.4%)	358 (59.8%)
Palliative Care	With palliative care	151 (25.2%)	222 (37.1%)
	Without palliative care	448 (74.8%)	377 (62.9%)
Paired patient/ caregiver	Non-paired	374 (62.4%)	374 (62.4%)
	Paired	225 (37.6%)	225 (37.6%)
Highest Education Level	Primary and lower	225 (37.6%)	122 (20.4%)
	Secondary and higher	314 (52.4%)	303 (50.6%)
	University	60 (10.0%)	174 (29.0%)
KPS Scores	KPS 0–40	100 (16.7%)	176 (29.4%)
	KPS 50–70	291 (48.6%)	275 (45.9%)
	KPS 80–100	208 (34.7%)	148 (24.7%)
Awareness of Cancer^a^	No	6 (1.0%)	
	Yes	582 (97.2%)	
	Unsure	11 (1.8%)	
Receive Chemotherapy^a^	No	311 (51.9%)	
	Yes	283 (47.2%)	
	Unsure	5 (0.8%)	
Receive Radiotherapy^a^	No	558 (93.2%)	
	Yes	34 (5.7%)	
	Unsure	7 (1.2%)	
Cancer Type^a^	Colorectal	121 (20.2%)	
	Lung	106 (18.0%)	
	Breast	92 (15.6%)	
	Gynaecological	38 (6.3%)	
	Head and neck	26 (4.3%)	
	Pancreas	26 (4.3%)	
	Kidney	26 (4.3%0	
	Upper gastrointestinal	22 (3.7%)	
	Liver	14 (2.3%)	
	Others	128 (21.3%)	
Relationship with Patient^b^	Spouse		258 (43.1%)
	Child		262 (43.7%)
	Parent		5 (0.8%)
	Sibling		38 (6.3%)
	Extended family (cousin, aunt)		32 (5.3%)
	Friend		2 (0.3%)
	Others		2 (0.3%)
Aware of Diagnosis^b^	No		4 (0.7%)
	Yes		591 (98.7%)
	Unsure		4 (0.7%)
	Don’t wish to say		0 (0.0%)
Aware of Incurability^b^	No		32 (5.3%)
	Yes		499 (83.3%)
	Unsure		59 (9.8%)
	Don’t wish to say		9 (1.5%)

^a^ Data was not collected from the caregivers

^b^ Data was not collected from the patients

Table 2 shows context of caregiving where only 30.1% of the FCs had previous caregiving experiences and only 7.7% received formal caregiving training despite 65.3% of the patients deeming themselves to require some assistance in their normal activities. Most FCs were involved in decision making (92.5%) and providing emotional support (98.8%).

**Table 2:** Context of caregiving

Caregiving Variables		n (%)
Stop work to look after patient	No	494 (82.5)
	Partially	26 (4.3)
	Yes, on unpaid leave	22 (3.7)
	Yes, resigned	57 (9.5)
Physical caregiving	No	343 (57.3)
	Partially^a^	122 (20.4)
	Yes^b^	134 (22.4)
Hours spent in providing physical care (if applicable)	Mean (SD)	3.1 (5.0)
	Median (range)	0.0 (0.0–4.5)
	Min - Max	0.0–20.0
Financial caregiving	Don’t wish to disclose	1 (0.2)
	No	225 (37.6)
	Partially^a^	191 (31.9)
	Yes^b^	182 (30.4)
Emotional caregiving	No	7 (1.2)
	Partially^a^	135 (22.5)
	Yes^b^	457 (76.3)
Decision making	No	45 (7.5)
	Partially^a^	403 (67.3)
	Yes^b^	151 (25.2)
Received formal caregiving training	No	553 (92.3)
	Yes	46 (7.7)
Previous experience in providing care for someone sick	No	419 (69.9)
	Yes	180 (30.1)

^a^ “Partially” referring to less than 50% involvement in caregiving role

^b^ “Yes” referring to at least 50% involvement in the caregiving role

### Needs of Cancer patients and their caregivers

Tables 3 and 4 show the top rankings of needs of items in each domain by patients and their FCs according to their mean score respectively. The items “I needed information about financial support for patients, either from government and/ or private organisations (e.g. support for medical expenses)” and “I needed help with my economic burden due to this illness (treatment costs, loss of income)” were ranked as the top items for the domain “information” and “practical support” by the patients. While the FCs ranked “I needed information about financial support for medical expenses either from government and/ or private organisations” (x̄=1.22) and “I needed the treatment to be near home for the patient” (x̄=1.06) are ranked as the top items for the domain “information” and “practical support” respectively. Overall, the item that had the highest need score in “information” domain for both patients and FCs was “financial support for patients, either from government and/ or private organizations.

**Table 3:** Top ranked mean score of individual CNAT question according to domain (Patients)

		Mean Score
Information		
1.	I needed information about financial support for patients, either from government and/ or private organisations (e.g. support for medical expenses).	1.05
2.	I needed information about palliative care services	0.79
3.	I needed guidelines or information about complementary and alternative medicine	0.64
4.	I needed information about correct diet (food to eat, food to avoid)	0.59
5.	I needed information about the current status of my illness and its future course	0.56
Psychological problems		
1.	I needed help with worries that I would become a burden to others around me	0.80
2.	I needed help with my concerns for the family	0.66
3.	I needed help with worries about treatment sequelae	0.62
4.	I needed help in coping with fear of recurrence	0.59
5.	I needed help with accepting role changes at home, at work and/ or in society after this illness was diagnosed	0.48
Practical support		
1.	I needed help with my economic burden due to this illness (treatment costs, loss of income)	0.86
2.	I needed treatment near my home	0.72
3.	I needed transportation services for getting to and from the hospital	0.49
4.	I needed accommodation services near the hospital where I was being treated	0.39
5.	I needed someone to help me with housekeeping and/ or child care	0.23
Physical symptoms		
1.	I needed help with lack of energy and/ or fatigue	0.65
2.	I needed help with trouble sleeping or oversleeping	0.58
3.	I needed help with pain	0.54
4.	I needed help with lack of appetite	0.48
5.	I needed help with diarrhoea or constipation	0.47
Hospital facilities & services		
1.	I needed rehabilitation medical services to help with functional recovery after treatment	0.54
2.	I wished for a short waiting period between the reservation and the doctor appointment	0.53
3.	I needed a designated hospital staff member who would be able to provide counselling for any concerns, and guidance with the course of my treatment, from the point of diagnosis to the period after the discharge.	0.51
4.	I needed an opportunity to share experiences or information with other patients (e.g. patient support groups etc)	0.39
5.	I wished to be treated in a pleasant environment	0.32
Health-care staff		
1.	I wished to be able to see the doctor in a quick and easy way when in need	0.63
2.	I wished my nurses to promptly attend to my discomfort and pain	0.38
3.	I wished my nurses to explain any treatment or care that was being given to me	0.32
4.	I wished my doctor to be easy, specific, and honest in his/ her explanation	0.32
5.	I wished sincere interest and empathy from my nurse	0.30
Social/ religious/ spiritual support		
1.	I needed help in finding the meaning of my situation and in coming to terms with it	0.23
2.	I needed help and support from people close to me (family, friends)	0.21
3.	I needed help with difficulties that arose in family relationships after this illness was diagnosed	0.16
4.	I needed help with difficulties that arose in interpersonal relationships after this illness was diagnosed	0.11
5.	I needed religious support	0.10

**Table 4:** Top ranked mean score of individual CNAT question according to domain (FCs)

		Mean Score
Information		
1.	I needed information about financial support for medical expenses either from government and/ or private organisations	1.22
2.	I needed guidelines or information about complementary and alternative medicine	1.04
3.	I needed information about caring for the patient (symptom management, diet, exercise, etc.)	0.97
4.	I needed information about the current status of the patient’s illness and its future course	0.93
5.	I needed information about tests and treatment that the patient receives	0.88
Health-care staff		
1.	I needed to see the doctor quickly and easily when in need	1.08
2.	I needed nurses to promptly attend to the patient’s discomfort and pain	0.81
3.	I needed the doctor to be clear, specific, and honest in his/ her explanation	0.71
4.	I needed cooperation and communication among health-care staff	0.68
5.	I needed the nurses to explain treatment or care given to the patient	0.67
Practical support		
1.	I needed the treatment to be near home for the patient	1.06
2.	I needed help with the economic burden caused by cancer	1.01
3.	I needed a transportation service for getting to and from the hospital	0.71
4.	I needed accommodation services near the hospital where the patient was being treated	0.52
5.	I needed assisted care in hospital or at home	0.49
Hospital facilities & services		
1.	I needed a designated hospital staff member who would be able to provide counselling for any concerns, and guidance with the course of the treatment, from the point of diagnosis to the period after discharge.	0.75
2.	I needed a space reserved for caregivers within the hospital	0.65
3.	I needed a visiting nurse service for home	0.60
4.	I needed guidance about hospital facilities and services	0.52
5.	I needed the opportunity to share experiences or information with other caregivers	0.49
Health & psychological problems		
1.	I needed help with concerns about the patient	0.93
2.	I needed help with feelings of vague anxiety	0.47
3.	I needed help with feelings of anger, irritability, or nervousness	0.39
4.	I needed help with depression	0.30
5.	I needed help with loneliness or feelings of isolation	0.24
Family/ social support		
1.	I needed help with patient over-dependence	0.52
2.	I needed help with my own relaxation and my personal life	0.51
3.	I needed help with difficulties in family relationships after cancer diagnosis	0.34
4.	I needed help with the patient’s lack of appreciation of the caregiving	0.29
5.	I needed help with difficulties in interpersonal relationships after cancer diagnosis	0.24
Religious/ spiritual support		
1.	I needed help in finding the meaning of my situation and coming to terms with it	0.34
2.	I needed religious support	0.24

Table 5 shows the total CNAT scores of FCs (20.5 ± 17.8) were higher than that of the patients (14.1 ± 14.7). Both patients and FCs had “information” and “practical support” in their top three domains of palliative care needs. The second highest domain of needs was “psychological problems” (16.4 ± 21.5) in patients and “health-care staff” (23.4 ± 26.5) in FCs.

**Table 5:** Overall difference of needs between cancer patients and their caregivers

Patients’ Domain	Mean (SD)	Caregivers’ Domain	Mean (SD)
Information	19.5 (22.6)	Information	28.9 (27.0)
Psychological problems	16.4 (21.5)	Health-care staff	23.4 (26.5)
Practical support	16.1 (20.6)	Practical support	23.4 (24.1)
Physical symptoms	13.3 (16.6)	Hospital facilities & services	19.1 (21.3)
Hospital facilities & services	12.6 (16.1)	Health & psychological problems	13.7 (17.3)
Health-care staff	11.0 (18.3)	Family/ Social support	12.7 (17.9)
Social/ Religious/ Spiritual support	5.4 (13.0)	Religious/ Spiritual support	9.7 (20.1)
**Total CNAT Score**	14.1 (14.7)	**Total CNAT Score**	20.5 (17.8)

Table 6 shows the difference of needs between cancer patients and their FCs in different clinical settings, with/without palliative care and across different range of KPS. Under clinical setting, the inpatients (19.2 ± 16.4) and their FCs (26.0 ± 19.0) tend to have higher needs than the outpatients (10.5 ± 12.1) and their FCs (14.7 ± 14.3). In terms of palliative care, higher total CNAT score was observed in both patients (16.6 ± 12.9 versus 13.3 ± 15.2) and their FCs (25.1 ± 18.6 versus 17.7 ± 16.7) who received palliative care. For patients with palliative care, both FCs and patients had high needs in “Information” and “Practical support” domains. In terms of patients’ KPS scores, patients with lower KPS scores tend to have higher needs. Patients with 0–40 KPS scores had the highest needs in the “psychological problems” domain followed by “practical support” domain.

**Table 6:** Difference of needs between cancer patients and caregivers

Patient	Mean (SD)				Caregivers	Mean (SD)			
Inpatient/ SOC Setting	Inpatient Setting (***n*** = 249)	SOC Setting (***n*** = 350)		***p***-value	Inpatient/ SOC Setting	Inpatient Setting (***n*** = 305)	SOC setting (***n*** = 294)		***p***-value
Information	23.7 (23.2)	16.6 (21.7)		< 0.001	Health and psychological problems	17.1 (18.4)	10.2 (15.2)		< 0.001
Psychological problems	22.2 (24.1)	12.4 (18.4)		< 0.001	Family/ Social support	16.1 (20.0)	9.2 (14.5)		< 0.001
Health-care staff	16.4 (21.6)	7.1 (14.4)		< 0.001	Health-care staff	30.2 (28.9)	16.3 (21.6)		< 0.001
Physical symptoms	19.0 (18.7)	9.3 (13.5)		< 0.001	Information	35.1 (27.8)	22.3 (24.5)		< 0.001
Hospital facilities and services	17.7 (18.6)	9.1 (12.9)		< 0.001	Religious/ Spiritual support	12.3 (22.6)	7.0 (16.6)		0.001
Social/ Religious/ Spiritual support	7.4 (15.9)	4.0 (10.3)		0.002	Hospital facilities and services	25.2 (23.1)	12.8 (17.1)		< 0.001
Practical support	23.2 (23.9)	11.1 (16.1)		< 0.001	Practical support	30.4 (25.8)	16.1 (19.6)		< 0.001
Total score	19.2 (16.4)	10.5 (12.1)		< 0.001	Total score	26.0 (19.0)	14.7 (14.3)		< 0.001
**With/ Without Palliative Care**	**With PC** **(*n*** **= 151)**	**Without PC (*n*** **= 448)**		***p*-value**	**With/ Without Palliative Care**	**With PC** **(*n*** **= 222)**	**Without PC** **(*n*** **= 377)**		***p*-value**
Information	21.1 (21.7)	19.0 (22.9)		0.327	Health and psychological problems	16.3 (17.2)	12.2 (17.1)		0.004
Psychological problems	18.8 (21.4)	15.6 (21.5)		0.112	Family/ Social support	15.0 (19.0)	11.4 (17.0)		0.017
Health-care staff	12.4 (17.5)	10.5 (18.6)		0.259	Health-care staff	29.1 (29.2)	20.0 (24.2)		< 0.001
Physical symptoms	16.5 (14.8)	12.2 (17.0)		0.006	Information	33.9 (27.3)	25.9 (26.3)		< 0.001
Hospital facilities and services	15.6 (15.9)	11.6 (16.1)		0.008	Religious/ Spiritual support	12.5 (22.8)	8.1 (18.2)		0.01
Social/ Religious/ Spiritual support	5.4 (11.6)	5.4 (13.5)		0.981	Hospital facilities and services	23.5 (22.2)	16.5 (20.4)		< 0.001
Practical support	21.6 (22.1)	14.3 (19.7)		< 0.001	Practical support	30.8 (26.2)	19.0 (21.6)		< 0.001
Total score	16.6 (12.9)	13.3 (15.2)		0.016	Total score	25.1 (18.6)	17.7 (16.7)		< 0.001
**KPS**	**KPS 0–40 (*n*** **= 100)**	**KPS 50–70 (*n*** **= 291)**	**KPS 80–100 (*n*** **= 208)**	***p*-value**	**KPS**	**KPS 0–40 (*n*** **= 176)**	**KPS 50–70 (*n*** **= 275)**	**KPS 80–100 (*n*** **= 148)**	***p*-value**
Information	23.0 (20.3)	19.6 (23.3)	17.7 (22.5)	0.151	Health and psychological problems	17.6 (17.8)	14.2 (18.4)	8.2 (12.4)	< 0.001
Psychological problems	27.3 (24.1)	16.0 (21.3)	11.8 (18.4)	< 0.001	Family/ Social support	17.4 (20.2)	12.9 (18.6)	6.7 (10.2)	< 0.001
Health-care staff	13.9 (18.1)	11.4 (18.9)	9.1 (17.5)	0.089	Health-care staff	29.1 (28.7)	23.3 (25.8)	16.8 (23.4)	< 0.001
Physical symptoms	21.5 (16.8)	13.0 (15.5)	9.8 (16.6)	< 0.001	Information	34.7 (27.0)	28.6 (27.2)	22.3 (25.2)	< 0.001
Hospital facilities and services	17.0 (16.0)	13.5 (16.4)	9.4 (15.1)	< 0.001	Religious/ Spiritual support	12.6 (22.9)	9.2 (19.3)	7.2 (17.5)	0.047
Social/ Religious/ Spiritual support	6.3 (14.7)	5.5 (12.5)	4.9 (13.0)	0.65	Hospital facilities and services	25.4 (22.9)	18.8 (21.2)	12.1 (16.8)	< 0.001
Practical support	24.1 (22.4)	17.6 (21.5)	10.3 (16.3)	< 0.001	Practical support	33.2 (24.5)	23.1 (24.7)	12.3 (16.3)	< 0.001
Total score	20.1 (13.9)	14.3 (14.6)	11.0 (14.3)	< 0.001	Total score	26.3 (18.2)	20.4 (18.0)	13.6 (13.9)	< 0.001

Univariable and multivariable regression analysis were performed to assess the association between characteristics and total CNAT scores for patients (Table 7) and FCs (Table 8). Multivariable analysis results showed that younger age, inpatient setting, male gender, paired patients and FCs, higher education level and lower KPS scores were associated with higher needs in both patients and FCs. In addition, “Aware of Cancer” was selected for patients and “Employment Status” was included in the multivariable model for FCs. Patients who were unaware of cancer had much lower CNAT total scores, but the sample size was very small (6 out of 599 patients). On the other hand, FCs who were unemployed had higher needs after adjusting for other variables in the multivariable model. In terms of Education, both patients and FCs with secondary (had at least 10 years of basic education) and higher and university education had higher needs compared to patients/ FCs with primary education and lower. FCs had highest unmet needs in the “University” category” whereas patients had highest unmet needs in the “Secondary and higher” category.

**Table 7:** Predictors of needs based on demographic data (Patient)

Univariable and multivariable linear regression analysis on CNAT Total Scores (Patient)						
Dependent: CNAT_Total		No.	Coefficient (95%CI)	P (wald)	Adjusted Coefficient (95%CI)	P (wald)
Age	[24,91]	NA	−0.33 (− 0.44 to − 0.22)	< 0.001	− 0.31 (− 0.42 to − 0.20)	< 0.001
Clinical Setting	Inpatient	249	1			
	SOC	350	−8.78 (−11.07 to −6.49)	< 0.001	−6.59 (−9.29 to −3.90)	< 0.001
Race	Chinese	495	1			
	Indian	25	0.38 (−5.51 to 6.27)	0.899		
	Malay	67	5.78 (2.04 to 9.52)	0.003		
	Others	12	0.79 (−7.60 to 9.19)	0.853		
Gender	Female	304	1		1	
	Male	295	0.90 (−1.47 to 3.26)	0.457	3.14 (0.91 to 5.36)	0.006
Employment Status	Employed	176	1			
	Unemployed	423	−1.30 (−3.89 to 1.29)	0.325		
Aware of Cancer	Yes	582	1		1	
	No	6	−9.22 (−21.05 to 2.61)	0.126	−11.30 (− 22.26 to − 0.34)	0.043
	Unsure	11	−5.99 (−14.76 to 2.79)	0.181	− 2.66 (− 10.81 to 5.48)	0.521
Receive Chemotherapy	Yes	283	1			
	No	311	−0.14 (− 2.52 to 2.24)	0.908		
	Unsure	5	−4.95 (−18.00 to 8.10)	0.456		
Receive Radiotherapy	Yes	34	1			
	No	558	−7.56 (−12.63 to −2.48)	0.004		
	Unsure	7	−10.86 (− 22.78 to 1.06)	0.074		
Palliative Care	With Palliative Care	151	1			
	Without Palliative Care	448	−3.33 (−6.04 to −0.62)	0.016		
Paired with Caregiver	Non-paired	374	1		1	
	Paired	225	0.55 (−1.89 to 2.99)	0.66	3.20 (0.91 to 5.49)	0.006
Education	Primary and lower	225	1		1	
	Secondary and above	314	3.05 (0.53 to 5.57)	0.018	3.19 (0.81 to 5.56)	0.009
	University	60	1.34 (−2.85 to 5.52)	0.53	1.80 (−2.16 to 5.77)	0.373
Cancer Type	Breast	92	1			
	Colorectal	121	−2.64 (−6.63 to 1.36)	0.195		
	Lung	106	−0.37 (−4.48 to 3.75)	0.861		
	Multiple primary	20	−4.85 (−11.98 to 2.27)	0.182		
	Others	260	−2.38 (−5.89 to 1.12)	0.182		
KPS	[20,90]	NA	−0.18 (−0.25 to − 0.12)	< 0.001	− 0.13 (− 0.20 to − 0.06)	< 0.001

**Table 8:** Predictors of needs based on demographic data (Caregiver)

Univariable and multivariable linear regression analysis on CNAT Total Scores (Caregiver)						
Dependent: CNAT_Total		No.	Coefficient (95%CI)	P (wald)	Adjusted Coefficient (95%CI)	P (wald)
Age	[21,81]	NA	−0.18 (− 0.29 to − 0.08)	0.001	−0.13 (− 0.24 to − 0.02)	0.022
Clinical Setting	Inpatient	305	1		1	
	SOC	294	− 11.23 (− 13.94 to −8.53)	< 0.001	−7.07 (− 10.31 to −3.84)	< 0.001
Race	Chinese	467	1			
	Indian	37	5.13 (−0.82 to 11.08)	0.091		
	Malay	84	1.54 (−2.59 to 5.67)	0.464		
	Others	11	5.84 (−4.79 to 16.46)	0.281		
Gender	Female	375	1		1	
	Male	224	1.92 (−1.02 to 4.86)	0.201	2.12 (−0.69 to 4.93)	0.14
Employment Status	Employed	358	1		1	
	Unemployed	241	0.36 (−2.54 to 3.27)	0.806	2.15 (−0.86 to 5.16)	0.16
Aware of Diagnosis	Yes	591	1			
	No	4	−1.98 (−19.51 to 15.54)	0.824		
	Unsure	4	−2.39 (−19.91 to 15.13)	0.789		
Aware of Incurability	Yes	499	1			
	No	32	−7.28 (−13.61 to −0.94)	0.025		
	Unsure	59	2.70 (−2.08 to 7.49)	0.268		
	Don’t wish to say	9	−2.64 (−14.33 to 9.05)	0.658		
Palliative Care	With Palliative Care	222	1			
	Without Palliative Care	377	−7.34 (−10.23 to −4.45)	< 0.001		
Paired with Patient	Non-paired	374	1		1	
	Paired	225	−8.42 (−11.29 to −5.55)	< 0.001	−3.98 (−7.00 to −0.97)	0.01
Education	Primary and lower	122	1		1	
	Secondary and above	303	3.81 (0.10 to 7.53)	0.044	4.51 (0.91 to 8.11)	0.014
	University	174	6.69 (2.60 to 10.78)	0.001	5.92 (1.68 to 10.15)	0.006
KPS	[10,90]	NA	−0.25 (−0.32 to −0.19)	< 0.001	− 0.12 (− 0.20 to − 0.04)	0.004

## Discussion

The results of this study show that patients with advanced cancer and their FCs have needs in all the 7 domains with different intensities. Since patients with advanced cancer suffer from several physical and psychological symptoms [34], it may be natural that they have more needs. However, in our study, FCs appeared to have more needs than patients (Table 5, higher mean scores in all domains). This result suggests that caregiving burden could be much higher than common general perception and that caregivers also have a lot of unmet needs. A study by Chang et al. [15] established that caregivers of terminal cancer patients were more burdened than patients.

The top needs were found in the domain of information for both patients and their FCs across all settings and irrespective of whether patients received palliative care. The need for information is well reported in the literature [8, 11, 27, 28, 35–37].

Information need related to financial support was positioned first by both patients and their FCs and assistance with economic burden caused by cancer was positioned second for patients and fifth for FCs respectively under the “practical support” domain. The financial concerns caused by cancer is well reported in the literature [11, 13, 17, 18, 35, 38–40] and financial distress in patients with advanced cancer is found to have a negative impact on their physical, emotional and social well-being [41]. Many of the patients in our study are elderly and unemployed, and many of their FCs are also unemployed. Findings also reveal that 17.5% of FCs’ work was affected due to caring for the cancer patients and of these, 9.5% resigned from their work. Literature reveals that many cancer patients and their FCs have difficulties maintaining work, which results in economic burden on the whole family [5, 11, 13, 16]. In Angioli et als’ [5] study on the economic burden among 172 FCs of patients with advanced ovarian cancer, the researchers established the mean cost for each caregiver was €10,981 annually. Overall, work productivity loss had a significant, direct relationship with anxiety, depression, disrupted schedule, and health problems, and caregiver perceived burden of financial problems. Their study did not take into account of the financial support that FCs provide to support their relatives’ medical care which is common in the Asian setting where filial piety and family harmony obligate family members to assist with the financial burden which is evidenced in our study that 62% of caregivers provided financial support to the patients. This might reflect significant financial burden experienced by cancer patients and their FCs in Singapore in the current healthcare system. As such, active efforts to explore the financial challenges and concerns confronting both patients and their FCs in order to reduce the direct and indirect economic costs related to cancer is warranted to guide policy making. In addition, providing and developing further information and support strategies for both patients and their FCs based on identified needs in the interim is urgently needed.

Both patients and FCs identified practical support as the 3rd top domain of needs. Both groups wanted treatment to be near patients’ home and needed transportation assistance for getting to and from the healthcare facility. The need for repeated visits for cancer treatment on an outpatient or inpatient basis makes distance an important issue with which the patients and their FCs must manage during the disease trajectory. Literature indicates that transportation is one of major barriers impacting healthcare access resulting in delayed or missed appointments or discontinuity in follow-up care, and affecting particularly those with lower income or the under/uninsured [42–44]. This may mean that transportation is a relatively high burden for both patients and FCs. Transportation need is usually under addressed by health care providers. Our findings demonstrate that in addition to financial assistance programmes, caregivers support programmes could include practical support aspects such as transportation services. Further studies are also needed to establish the factors that make transportation a barrier, the impact of transportation barriers and the types of interventions needed.

There is also a high level of needs in the health care staff domain identified by FCs across all settings as compared to patients. FCs want to see their doctor quickly and easily when needed. In our study, 93.5% of FCs were involved in decision-making and this role inherently requires readily available access to the patients’ doctor. The availability and accessibility of health care staff remains an important area of health service delivery, especially in the context of an increasing complex medical treatment environment [45]. A study of caregivers’ needs in Korea established this as the top need [27] and another study in Iran also found out that health care staff need is the second commonest demand reported by caregivers [28]. Results reflected the gap between health care services provided and caregiver’s experiences on service delivery. Our findings also suggest that patients are relatively passive in the decision-making process and as such, it is necessary to include FCs during the treatment making process with the patients. This is supported by a study conducted in an Asian setting, where 97.2% of family members made decisions for patients [46].

Our study also revealed the psychosocial impact affecting the cancer patients (3rd top scores) and almost all (98.8%) of FCs provide emotional support to the patients. This is on top of their need for help with concerns about the patients (top 6 need) and help with their own emotional feelings. Consistent with previous findings, patients and their FCs experienced a lot of emotional distress during the cancer trajectory and especially so for those at the advanced cancer stages [8, 25, 47, 48]. Given the psychological impact of cancer on patients and their FCs, understanding and addressing their psychological and emotional needs is needed in order to improve their well-being.

Both patients and FCs identified their need for guidelines or information about complementary and alternative medicine (CAM), as the 4th most important need. The interest in the use of CAM is not unexpected in the cancer trajectory especially when confronting the advanced stage or terminal stage. The hope for cure and the easily accessibility of CAM products may prove irresistible to these vulnerable patients and FCs. Surveys conducted in Singapore confirmed the interest in information relating to CAM [35, 36]. A study conducted in Singapore showed high prevalence of CAM use (55%) among cancer patients especially those with stage 4 disease while receiving chemotherapy or radiotherapy. Thirty-seven percent of patients believed CAM to be equally or more effective than conventional treatments [49]. However, the use of CAM in combination of conventional therapies is not without risks [50, 51], and therefore is a valid need that warrants attention.

In our study, we found that patients who received palliative care and their FCs have more needs than those who did not receive palliative care. Although, it can be expected that those who received palliative care are more ill (tended to be inpatients and lower KPS) and therefore have more needs, as early palliative care has been proven to improve the QOL of the patients with advanced cancer [52–56], we postulate that this may also imply that patients with advanced cancer were referred by their oncologists to palliative care physicians late in their cancer trajectory. This late referral may have resulted in patients who are more ill and therefore, have more needs and require more care. The findings that almost 75% of the patients group and almost 63% of the FCs group cared for patients who were not referred to palliative care physicians in spite of their advanced stage in cancer, and patients have also identified under the domain on “Information” as the top second information needs as “I needed information about palliative care services” may add credence to this view. In order to ascertain the real reason why patients and their FCs who received palliative care have more needs, further study would be needed as late referral to palliative care is associated with aggressive end of life treatment [55].

Our study also revealed that the lower the KPS scores, the higher level of needs for both patients and their FCs. We postulate that because patients with lower KPS scores are less able to provide self-care due to their decreased functional abilities, they are more dependent on others to assist them in the activities of daily living (ADL), they tend to have more needs and those providing care to them will inadvertently have also more needs. Similarly, both inpatients groups of patients and their FCs have more needs as compared to outpatients. Mawardika et al. [57] reported that gynaecological cancer patients’ physical and psychological supportive care is comparatively higher (44 times) for those receiving inpatient care than those receiving outpatient care. Besides, the financial cost associated with hospitalization is considerably higher than outpatient and as such, they logically have more needs. We also found that older patients tended to have more needs in the inpatients group. It may be that older patients tended to be more physically challenged and less likely to be employed, and therefore have more needs. In addition, female inpatients tended to have more unmet needs. The reason is unclear and the possible explanation being the traditional roles Asian women play in supporting the family. Being hospitalized may impact their ability to perform their functions well.

Our findings provide important information to assist in the identification of at-risk groups based on socio-demographic characteristics of both patient and their caregivers who warrants more attention.

### Practice implications

Our study has important implications from both clinical and research perspectives. From the clinical perspective, findings from this study elucidate areas to attend to in clinical practice and the need for healthcare professionals to systematically assess patients’ and FCs’ problems (including caregivers’ training needs as part of quality care for patients) and provide timely information and supportive care. In addition, our results highlight the importance of ensuring sufficient resources are allocated to the development of appropriate strategies such as providing and developing further information materials and support programmes and services to address the key areas of identified needs of patients with advanced cancer and their FCs. From a research perspective, our results suggest that more studies need to be done to establish the financial challenges and concerns confronting both patients and their FCs in order to reduce the direct and indirect economic costs related to cancer. In addition, as early palliative care has been proven to improve the QOL of the patients with advanced cancer, it is important to ascertain the reasons for late referral to palliative care. Finally, a periodic audit of the needs of patients with advanced cancer and their FCS and how well their needs are met should be conducted under a patient and family centered approach in order to understand and address their unique and evolving needs.

### Limitations

Our study has some limitations. This was a cross-sectional study at a particular point in time does not describe the longitudinal trends in needs in the course of the disease trajectory. An additional limitation was the non-randomized nature of sampling resulting in the possibility of selection bias as only patients and FCs who agreed to participate were recruited. Moreover, data on non-respondents were not systematically collected and as such, the participants may not be representative of the general population of patients with advanced cancer and their FCs. Despite these limitations, our study was conducted in the largest public ambulatory cancer centre and the largest public hospital in Singapore and the large sample size with equal number of patients and FCs, with the use of standardized and validated questionnaires for both patients and their FCs should have mitigated these limitations. Additionally, we were also able to demonstrate the differences in needs based on sample (patients vs FCs), settings (inpatients vs outpatients), care received (palliative vs non-palliative), and KPI scores. Thus, we believe that the results can be generalized to other settings.

## Conclusion

In summary, our study revealed that both patients and their FCs have many needs irrespective of their clinical settings. The top needs were found in the domain of information for both patients and their FCs across all settings and irrespective of whether patients received palliative care. The top need for both patients and their FCs relates to information about financial support suggesting the financial burden they are bearing and an area that warrants attention and support both by healthcare providers and the policy makers. In addition, FCs also tended to have more unmet needs than patients especially in the area of practical, emotional and psychological support, and quick access to healthcare professionals.

This study contributes to the growing literature of needs of patients with advanced cancer and their FCs and suggests that in order to deliver quality care to cancer patients, it is important to ensure that their FCs’ needs are also not neglected.

## Data Availability

The datasets used and/or analysed during the current study available from the corresponding author on reasonable request.

## Abbreviations

FC: Family caregivers
CNAT: Comprehensive Needs Assessment Tool
CNAT-C: Comprehensive Needs Assessment Tool for Cancer Caregivers
KPS: Karnofsky Performance Status
ANOVA: Analysis of variance
AIC: Akaike Information Criterion
CAM: Complementary and alternative medicine
ADL: Activities of daily living
